# “It's ok if it's hidden”: The discursive construction of everyday racism for refugees and asylum seekers in Wales

**DOI:** 10.1002/casp.2344

**Published:** 2018-03-09

**Authors:** Samuel Parker

**Affiliations:** ^1^ School of Social Sciences Cardiff University Glamorgan Building Cardiff CF10 3WT UK

**Keywords:** asylum seekers, discourse analysis, discursive psychology, integration, racism, refugees, Wales

## Abstract

Wales has a long history of migration; however, the introduction of dispersed asylum seekers in 2001 has led to Wales becoming a more superdiverse nation. Wales has often been positioned as a more “tolerant nation” than England; however, the increasingly superdiverse nature of Wales in a postdevolution era may now be calling this tolerance thesis into question. Models of refugee and asylum seeker integration suggest that the absence of racism plays a key role in integration. This paper reports the findings of research that centres on refugee and asylum seeker integration in Wales. Nineteen interviews were conducted with refugees and asylum seekers who had been living in Wales for between 1 month and 12 years. Each interview was analysed using a discursive psychology approach. In this paper, I show that the interviewees appeared to negotiate a dilemma when talking about experiencing potentially racist incidents within the interviews, constructing them as trivial so as not to appear critical of the protection they have received in Wales. The findings also highlight the more everyday and banal forms of racism that are regularly experienced by refugees and asylum seekers living in Wales.

## 1 INTRODUCTION

This article reports on an analysis of talk by asylum seekers and refugees living in Wales with regard to their experiences of discrimination and racism. It builds on work published in a recent special issue of this journal by extending the focus from media representations of refugees (Goodman, Sirriyeh, & McMahon, 2017) and of lay discourses (Nightingale, Quayle, & Muldoon, 2017), to the discourses of refugees and asylum seekers themselves, and the ways in which they talk about their experiences of discrimination and racism. The development of research into prejudicial or racist talk has been one of the key contributions of the discursive psychological approach stemming from the work of Wetherell and Potter (1992) and Billig (1988, p. 94), who coined the phrase the “norm against prejudice” to describe his finding that speakers can go to great lengths to rhetorically distance themselves from accusations of prejudice. Augoustinos and Every (2007) extended Billig's work further and identified a number of discursive strategies employed by speakers to avoid violating this norm. These included denials of prejudice (“I'm not racist, but …”), the grounding of views as reflecting the external world, positive self and negative other presentation, and discursive deracialisation (the removal of race from discourses that could potentially be seen as being about race).

Although there has been a large body of research focusing on constructions of forced migrants in the media (Baker & McEnery, 2005; Goodman et al., 2017; Lea & Lynn, 2003), in political speeches (Capdevila & Callaghan, 2008; Charteris‐Black, 2006; Every & Augoustinos, 2008) and in lay discourses (Goodman & Burke, 2011; Kirkwood, McKinlay, & McVittie, 2015), more recent research has taken forced migrants' discourses themselves as the topic of study. Kirkwood, McKinlay, and McVittie (2013) carried out semistructured interviews with 15 asylum seekers living in Glasgow. They found that bringing up the topic of racism was a strategy that had to be employed “delicately” (Every & Augoustinos, 2008; Goodman & Burke, 2011) and only as a last resort, in such a way as to deny that they may be making an accusation of racism. This strategy was often employed by reducing the seriousness of racially motivated violence or denying that the incident was indeed racist so as to avoid negative evaluations of the host society. In situations where the speaker did attribute violence to racism, a “delicate” strategy had to be employed once more, with speakers using strategies such as attributing the violence to the ignorance of the attacker, so as not to appear “… overly sensitive to racism” or be seen to be making complaints (Kirkwood et al., 2013, p. 758). Similarly, Goodman, Burke, Liebling, and Zasada (2014) found that whilst participants criticised the U.K. asylum system and said that they were unhappy in the United Kingdom, a dilemma was created for them that risked undermining the reason they were claiming asylum in the United Kingdom and appearing ungrateful. Goodman et al. therefore report a downplaying of not being happy as a means of resolving this dilemma, much like the downplaying of “racism” seen in Kirkwood et al.'s (2013) study.

The present study focuses on forced migrants in Wales, UK, which has a long, but often overlooked, history of migration. Evans (2015) draws attention to Irish and Jewish migrants settling in Wales in the late 19th century, to black seamen settling in Cardiff Bay in the early 20th century, and to a wave of migration from Italy following World War II. However, Payson (2015) has noted that when the United Kingdom as a whole has accepted large quotas of refugees in response to humanitarian crises, such as Bosnians or Rwandans in the 1990s, Wales was largely unaffected by such immigration. Similarly, only a few of the thousands of Vietnamese resettled in the United Kingdom in the 1970s were located in Wales. More recently, however, Wales has once again seen increases in immigration: first, as a result of the opening up of the U.K. labour market to A8 EU migrant workers in 2004 and second, dispersal of asylum seekers to Wales began in 2001 following the 1999 Immigration and Asylum Act. In order to receive subsistence and accommodation support under Section 95 of the Act, asylum seekers were required to accept accommodation wherever this was available. As such, Cardiff, Swansea, Newport, and Wrexham all became “dispersal” areas in 2001 and marked, for the first time, any significant arrivals of asylum seekers to Wales. Home Office (2017) data reveal that at the end of December 2016, there were 3,009 asylum seekers (main applicants, supported under Section 95 of the Immigration and Asylum Act, 1999) living in Wales and that the percentage of asylum seekers being dispersed to Wales has increased from 4.6% of total U.K. applications in 2005 to 7.6% in 2016. Crawley (2013) further estimates that there may be between 6,000 and 10,000 refugees also living in Wales.

Vertovec (2007, p. 1025) suggests that the United Kingdom can be characterised as “superdiverse,” arguing that it is not enough to see diversity only in terms of ethnicity, as is regularly the case both in social science and the wider public sphere. Such additional variables include differential immigration statuses and their concomitant entitlements and restrictions of rights, divergent labour market experiences, discrete gender and age profiles, patterns of spatial distribution, and mixed local area responses by service providers and residents. In a review of ethnic diversity in Wales, Williams (2015) suggests that the increasingly superdiverse nature of Wales has led to increased challenges to the “Tolerant Nation” thesis, which proposed that Wales' national character was both egalitarian and welcoming to immigration and positioned Wales as more accommodating of difference and diversity than other parts of the United Kingdom. Although she draws attention to the fact that many areas of Wales have not been characterised by large‐scale immigration, in her criticism of the tolerance thesis, Williams (2015) discusses the challenges to Welsh national identity, particularly from inward migration from England. Indeed, she suggests similarities between black oppression, the popular imagining of Welsh oppression by the English, and that the tolerance thesis has focused too greatly on English migration at the expense of exploring its relevance for racialized minorities.

Academic research into the settlement experiences of ethnic minorities in Wales is limited, although research in this area has increased since the dispersal of asylum seekers to Wales began in 2001. Threadgold et al. (2008, p. 3) suggest that “there is a significant history of racism which does not always sit easily beside the myth of warm, accepting proletarian Wales.” Indeed, Crawley and Crimes (2009) report that over half of all refugees in Wales have experienced racism or negative attitudes towards them.

In the present paper, I begin by outlining the methods used in this study. I then proceed to explore how refugees and asylum seekers talk about discrimination and racism that they report to have experienced whilst living in Wales. I examine the functions of this talk and consider the implications of these within the wider context of refugee and asylum seeker integration in a changing Wales.

## 2 METHOD

### 2.1 Data

The data analysed in this study come from 19 individual semistructured interviews conducted with refugees and asylum seekers who were living in Wales at the time of the interview. Participants were invited to take part in the interviews to discuss their experiences of integration and living in Wales. They were recruited from refugee and asylum seeker support organisations in Cardiff and Swansea. A total of 11 male and 8 female participants took part in this study who ranged in age from 19 to 58, with an average age of 34. Participants had been living in the United Kingdom for between 1 month and 12 years at the time of interview, with an average time in the United Kingdom of 40 months, and were from 13 different countries of origin. Four of the participants were asylum seekers who had made an initial application for protection to the U.K. Government, seven were asylum seekers whose case had been refused by the U.K. Government and who were appealing the decision at the time of interview, seven participants had been recognised as refugees and granted 5‐year leave to remain in the United Kingdom, and one participant had been granted British Citizenship. Each of the participants had been initially sent to Wales on a no‐choice basis by the U.K. Home Office in order to claim Section 95 support under the 1999 Immigration and Asylum Act. Those with refugee status had chosen to remain in Wales following their grant of status.

All participants were informed that the interviews would be conducted in English and that they needed to have sufficient English speaking and listening skills in order to discuss their experiences of living in Wales. All interviews were conducted in English except for one that was conducted partly with the aid of a translator. Interviews were audio‐recorded and transcribed using a simplified version of the conventions outlined by Jefferson (2004), each lasted between 18 and 62 min, with an average length of 32 min. Semistructured question guides were used with questions relating to each of the 10 domains identified in Ager and Strang's Indicators of Integration framework (Ager & Strang, 2004) and included only one question that directly asked participants whether they had experienced any forms of prejudice or racism whilst living in Wales. Each of the interviews were initially coded using NVivo in order to identify sections in which prejudice or racism was raised by either the participants or the researcher. This resulted in eight extracts, where prejudice or racism was reported by the interviewee, being selected for further detailed analysis. Therefore, 11 of the participants did not report experiencing prejudice or racism in the interview.

### 2.2 Analytic approach

In this analysis, I have adopted a discursive psychological approach, which treats language as a form of social action. McKinlay and McVittie (2008) suggest that discursive psychology emerged in order to provide a more naturalistic and functional approach to the topics classically studied within social psychology. Discursive psychologists, such as Wetherell and Potter (1992), criticised the dominant, cognitive, approach within social psychology, arguing that such approaches could not account for the variation they had found in their interview participants' talk. As a result, discursive psychology is concerned with people's practices, in particular what they are doing with their discourses in terms of argument, communication, and interaction in specific, situated, settings. In this way, discursive psychology can be distinguished from forms of discourse analysis in other fields (such as in linguistics), which analyse the grammatical and stylistic organisation of discourses, by placing greater emphasis on language use and what is achieved by that use. Analysis of the eight selected extracts revealed that three participants constructed accounts that made the incident they described appear trivial. Analysis of a further three extracts revealed that although participants denied experiencing prejudice or racism in Wales, their accounts constructed racism as something which is “hidden” and therefore unproblematic for them.

## 3 ANALYSIS

In this section, I present an analysis of extracts from the interview data, which together illustrate two distinctive ways in which refugees and asylum seekers living in Wales constructed accounts of incidents in which they suggest they may have experienced personal discrimination, constructing incidents of discrimination as trivial and banal “hidden” forms of everyday racism. In both cases, I demonstrate how the construction of such accounts may be dilemmatic for the participants, which is contingent on their precarious status.

### 3.1 Constructing incidents of discrimination as trivial

Previous discursive research with asylum seekers and refugees in the United Kingdom (Goodman et al., 2014; Kirkwood et al., 2013) has pointed to the ways in which participants may downplay incidents of discrimination when giving personal accounts of their experiences of living in their new communities. Notably, although the data in the current study come from interviews that asked questions about a range of integration topics (based upon Ager & Strang's, 2004, Indicators of Integration model), examples of discussing discrimination were not limited to when participants were asked direct questions about whether they had experienced discrimination.

In Extract 1, Amna,^1^ who had been living in Wales for 12 years at the time of interview and had been granted British Citizenship, is initially hesitant to answer the question posed by the interviewer (Sam) in Lines 1 to 3.

Extract 1 **2Amna**

1 Sam: since you've been living in Cardiff do you feel like

2 you have experienced any‐ anything negative

3 any prejudice or °discrimination°?

4 Amna: (2.0) well‐ (.) Sometimes yes I can see but

5 I am a positive positive person heh heh heh

6 I try to be positive in the society but er yeah

7 sometimes (.) maybe teenagers people they they

8 teasing on somebody like me

9 Sam: Mm mm

10 Amna: because they‐ I am a foreigner (.)

11 >I am British now< but I am a foreigner it's not‐

12 honestly that's what it is (.) but err hh but

13 some years ago they tried to (.) just throw stones

14 to me and hh I tried just to ignore it it's it's not easy

15 it's not easy honestly but I try especially I was

16 very angry (.) because not of them they are they

17 were teenagers ok they were teenagers

18 but they mum (.) were with them

19 Sam: oh

20 Amna: is makes different

21 Sam: yeah

22 Amna: If they were just teenager that's ok‐

23 >it's not ok< but I can (.) cope with that

24 Sam: mmm

25 Amna: because it's ok they are child no problem

26 but they mum were with them and

27 they didn't say anything to them

[10 lines omitted]

28 Amna: no no no none of them three (.) mums

29 with children none of them (.) didn't say anything

30 >just a stop< for me it's not good to do that

31 or not DON'T do that nothing nothing at all

32 Sam: but things like this have only happened‐

33 Amna: it's you know it's you know when (0.5)

34 mum says nothing looks‐ ok this is ok that attitude

35 is ok to do in the society which it's not

36 Sam: but this has only happened once?

37 Amna: just a °couple of times°

The question, through its use of “anything negative” and “any prejudice or discrimination,” is put to Amna as an extreme case formulation (ECF; Pomerantz, 1986). Heritage and Robinson (2011), in their study of clinician and patient interactions, show that use of the term “any” in questions has negative polarity and is therefore geared towards negative responses. Constructing his ECF with three uses of “any” allows Sam to put into play a question that orients to the idea that there is nothing unusual in experiencing negative or discriminatory incidents, therefore making it more difficult for Amna to make a denial. Sam's question also offers Amna three broad and inclusive candidate “experiences” to consider. Amna's hesitations are shown in the 2‐s pause beginning her turn in Line 4 before continuing with the discourse marker *well*. Jucker (1993) has reviewed a number of possible uses of this marker and suggests that it can have a number of functions: as a marker of insufficiency, as a face‐threat mitigator, as a frame, and as a delay device. Jucker (1993) suggests that use of *well* as a marker of insufficiency is a device used by respondents who know that they are not giving the information requested by the questioner. This explanation for use of the marker *well* appears to be borne out by Amna's subsequent talk in Lines 4 to 7 in which she constructs herself as a positive person. However, this talk also suggests that *well* may be used here as a face‐threat mitigator and as a delay device. This strategy of constructing herself as a positive person also highlights a reluctance on her part to make an accusation of discrimination. Indeed, it is not until Lines 7 to 8 that a suggestion of discrimination is raised.

Interestingly, in Line 8, Amna first raises the discrimination experienced as “teasing” suggesting that the explanation for this incident could be related to the age of those involved. The word “teasing” is itself one that has childhood connotations and is employed to explain the incident. In doing so, it makes the incident appear trivial by claiming that it is something we would expect from people who are young. In Lines 10 to 12, however, a competing explanation is put forward to explain the incident, Amna's identity as “a foreigner.” This suggests that the behaviour was racially motivated and, due to attributes, related to her. “Honestly that's what it is” (Line 12) further suggests that she was reluctant to choose this over other possible explanations and allows Amna to avoid making general accusations of racism. Further details of the incident, in which stones were thrown at Amna, are not reported until Lines 13 to 14, following Sam's use of the discourse continuer (“mm mm”) in Line 9, which prompts Amna to say more about the incident. In Lines 13 and 14, the discourse marker “just” is used as a means of constructing the incident as trivial (“just throw stones”). Weltman (2003) shows that, in the discourses of politicians, use of the discourse marker “just” can function to describe an event as happening virtually accidentally. Such a strategy is employed here and allows Amna to put an explanation into play without attributing blame and avoids the negative consequences associated with making claims of discrimination (Kirkwood et al., 2013).

However, in Lines 15 to 18, the parents of the teenagers are constructed as being responsible for the incident because they were present at the time. This portrays Amna as having an issue with the mother, and her parenting, rather than with the teenagers, and their racism. This dilemma is further shown in Lines 22 to 27 where Amna initially states that it was okay because the perpetrators were teenagers before quickly repairing this to say that it is not okay but that she can “cope with that.” This again implies that she cannot cope with the mothers allowing such behaviour from their children. Further rhetorical work is achieved in Lines 28 to 35 where Amna provides further justifications for her view that responsibility for this incident lay with the parents not stopping their “children” (switched from teenagers in Line 22) from engaging in this behaviour. Again, this provides further justification for the event being a form of everyday racism, which is to be expected from children, if their parents do not teach them what is right and wrong in society. Her focus on the parents functions to portray the incident as problematic, and the speaker as being concerned with it, whilst allowing Amna to position herself as not making an accusation of racism as such, as being “overly sensitive,” or having a problem with all members of the local society (Kirkwood et al., 2013).

Munir, in Extract 2 below, also describes an incident that could be classed as everyday racism, which is also constructed as trivial through drawing on the age of those involved in the incident and a description using generalised and vague terms.

Extract 2 **Munir**

1 1 Sam: you've not had any problems (.) with any people?

2 Munir: never I don't find any

3 Sam: no::

4 Munir: sometimes you see in the evening some guys

5 or (.) some er young guys it's up to the just playing

6 with every (.) everybody (.) not er=

7 Sam: yeah

8 Munir: especially asylum but er (0.5) no problem for us

9 Sam: yeah

10 Munir: but from the (.) adults and er I don't find any problem

11 Sam: ok

12 Munir: they::‐ sometimes they er help us (.) yeah they are

13 very kind with us I don't find any problem

Sam's questioning here follows the same strategy employed in the previous extract through the use of “any problems” and “any people,” which may presuppose a negative response (Heritage & Robinson, 2011). In contrast to the previous extract that used a number of hedged constructions, Munir begins his turn in Line 2 with two ECFs (“never” and “I don't find any”) that function to deny the existence of “any problems.” However, in his second turn (Lines 4–6), we see a contradiction where he constructs an account of everyday racism perpetrated by “young guys” who are “just playing.” As play is something particularly associated with children, and those who are “young,” it suggests that if they are “just playing,” it is relatively trivial and can perhaps be considered as “nondiscrimination.” Similarly, a disclaimer is also used in Lines 6 and 8, which suggests that these young people were not specifically targeting asylum seekers, but “everybody.” This ECF functions to normalise the behaviour and portray it as not necessarily racist because it was directed at “everybody,” not just asylum seekers. In Line 10, we see that this claim is upgraded so that it is only children who are constructed as problematic and at the end of the extract there is a further upgrade to “I don't find any problem.” By locating the problem with a small number of people, and not others, Munir's talk functions to portray him as not having a problem with all members of the local population, thereby managing the implications of making accusations of racism.

In both of the extracts discussed thus far, I have demonstrated that, in line with previous work (Goodman et al., 2014; Kirkwood et al., 2013), when accusations of discrimination are made by asylum seekers and refugees in Wales, they are made to appear trivial which avoids making general negative evaluations of the host society. In the next section, however, I present another way in which participants oriented to questions about discrimination, questioning whether incidents of discrimination they had experienced could be attributed to “race.”

### 3.2 Banal “hidden” forms of everyday racism

In Extracts 1 and 2, I suggested that the described incidents of racism were constructed as trivial in order to avoid negatively evaluating the host society. However, in Extract 3, Kris, who had been living in the United Kingdom for approximately 8 months at the time of the interview and had recently been granted refugee status, also constructs a trivial account, but one in which he “senses” that he may have encountered discrimination in Wales.

Extract 3 **Kris**

1  Sam: have you experienced any (.)
2: prejudice or racism discrimination?
3  Kris: not as yet
4  Sam: no
5  Kris: not as yet (.) yeah (.) every place I've
6: gone to like hh (.) yeah
7: >you meet different people< and people
8: >you know< people behave differently
9: but at least with racism I haven't found
10: that yeah
11  Sam: good
12  Kris: somebody might not talk to you
13  Sam: yeah
14  Kris: but he hasn't mentioned anyone‐ any‐
15: anything to do with the colour or something
16: so yeah you can sense what it is but err
17: at least nobody has spoken to that or nobody
18: has turned me down because of my colour
19: or anything

Sam's question (Lines 1–2) gives three candidate “expressions” for Kris to consider and again uses the more negatively polarised “any” interrogative form (Heritage & Robinson, 2011). In Line 3, Kris states that he has not experienced any discrimination whilst living in Wales, which he repeats again in Line 5. However, in making this denial, he uses the hedged response “yet” in both cases. Use of this hedging device may indicate that racism and discrimination are to be expected in Wales and that Kris may be experiencing a dilemma in discussing such issues. Indeed, as the extract continues, we see this dilemma playing out further. In Lines 5 to 10, Kris begins to hesitate (indicated by the pauses) and then constructs an account in which a question asked about racism and prejudice is turned into one of people behaving differently and is initially dismissed as being related to “race.” His use of “people behave differently” (Line 8) is a more general statement that may function to normalise racism and variation in behaviour. As his account continues, Kris (in Lines 14 to 19) describes how he can “sense” that he feels he might be treated differently because of his skin colour; however, the reference to colour remains ambiguous and is not raised until Line 15. This ambiguity could suggest that nobody has been racist or that they may indeed have been influenced by prejudice. Because there is no explicit reference to “colour,” in the situation Kris describes, it could therefore be that “people behaving differently” is less bad or definitely not racist. This again suggests that everyday racism could be at play here, albeit not in the forms discussed in the previous extracts.

In Extract 4, Mustafa, a refused asylum seeker who had been in Wales for 1 year at the time of interview, constructs a similar account to that of Kris, which works to suggest that “hidden” racism may be a feature of his everyday encounters with British nationals in Wales.

Extract 4 **Mustafa**

1

1  Mustafa: some people told me some people here .hhh
2: err racist with us er racist with us but er for me
3: I (1.0) I didn't see anything like this
4  Sam: mmm you've never experienced any (.)
5: racism here?
6  Mustafa: no no I can feel it but (.) nobody .hhh err:::
7: say something bad for me you know? (1.0) .hhh
8  Sam: when you say you can feel it (.)
9: what‐ how do you?
10  Mustafa: no it's ok if he if he if he ignore it so or for
11: he hidden it .hhh so it's ok for me (.) yea:::h
12: but if he show it to me I will(1.0) I will be a
13: different person heh heh I think so heh heh

Mustafa raises the issue of racism in Line 2 by suggesting that it is something that he has heard from other people but denies “see (ing) anything” himself (Line 3). Such a strategy allows Mustafa to put the suggestion of racism occurring in Wales into play but avoids making direct accusations of racism. In Lines 4 and 5, Sam responds to Mustafa's previous utterance, seeking clarification through the use of a negatively polarised question (Heritage & Robinson, 2011) that is constructed as an ECF. In Line 6, Mustafa makes a second clear denial of experiencing racism but immediately repairs this by saying “I can feel it,” which functions in a similar way to Kris's use of “you can sense what it is” in Extract 3. His use of “you know?” (Line 7) also implies that this is an everyday experience that Sam should understand. However, Sam's subsequent response suggests that it is not a shared understanding because he asks for further explanation rather than agreeing with Mustafa's previous question. Mustafa's final turn is marked by a number of hesitations and repetitions that suggest he may be uncomfortable accounting for his previous turn. Here, he constructs an account in which “hidden” racism is acceptable to him but more explicit forms of racism are not. Indeed, he suggests that he would only consider it problematic “if he show it” (Line 12). Thus, racism here, as in Extract 3, is constructed as something that is invisible but which has the potential to become visible. In the next section of this paper, I discuss the implications of this further within the context of refugee and asylum seeker integration in Wales.

## 4 DISCUSSION

In this paper, I have taken a discursive psychological approach to study how accounts of discrimination and racism are constructed by forced migrants living in Wales. The analysis has identified two ways in which the asylum seekers and refugees in this study constructed accounts of discrimination, which they report to have experienced whilst living in Wales. These will now be discussed in relation to forced migrants' integration in Wales and the claims that the increasingly superdiverse nature of Wales challenges the tolerance thesis.

Analysis of the extracts presented in this paper has shown that participants either constructed the reported incidents of discrimination as trivial or constructed accounts where banal everyday encounters gave them the feeling that they may have experienced racism in Wales. There is evidence in the extracts that such complaints share features with other forms of talk where complaints are raised reluctantly or only through necessity (Edwards, 2005). Indeed, the complaints raised in Extracts 1 and 2 pursue this strategy in order to put the experience of discrimination in play whilst avoiding the negative consequences of making accusations of racism (Kirkwood et al., 2013). It is suggested that such strategies also allow the speaker to avoid complaining about the society and appearing ungrateful for the protection which they have received, in line with the findings of Goodman et al. (2014) and Kirkwood et al. (2013).

Although Kris and Mustafa also constructed discrimination as being trivial to some extent, their strategy of questioning the basis of the discrimination again allows the explanation of racism to be put in play but also avoids some of the difficulties that can follow from making direct accusations of racism. Flam and Beauzamy (2008) draw on Billig's (1995) argument relating to banal forms of everyday nationalism to develop an account of how migrants are confronted by different forms of rejection in their everyday encounters with natives. Also drawing on Essed's (1991) notion of everyday racism, they suggest that “rejection takes many forms, ranging from rendering one invisible to negative singling out, from averted gaze to bodily attack and outright violence” (Flam & Beauzamy, 2008, p. 223). Both Kris and Mustafa constructed accounts that support the idea of everyday racism, a sense of feeling discrimination in everyday encounters that are not explicit as those discussed in the specific examples by Amna and Munir.

Extract 4 from an interview with Mustafa is of particular relevance because of his status as a refused asylum seeker, which is in contrast to those in the first three extracts who had all been granted refugee status in the United Kingdom. His status might lead us to hypothesise that his reluctance to make accusations of racism may be as much to do with the asylum system itself as it is to do with an unwillingness to criticise the host society. Bloch and Schuster (2005) have shown how changes to asylum policy under the New Labour government in the United Kingdom from 1999 onwards sought to create a “hostile environment” in which policies of dispersal, destitution, detention, and the deportation of asylum seekers were introduced as a means of deterring people from entering the United Kingdom in order to claim asylum. Similarly, Sales (2002) is critical of the U.K. asylum system, which she claims is predicated on the notion that all asylum seekers are bogus and “undeserving” of either entry to the United Kingdom or social support. Experiencing a system where you are not believed may therefore explain reluctance to make direct accusations of discrimination, which may both seem critical of the host country and potentially undermine the reasons why someone is seeking protection. Indeed, Goodman et al. (2014) suggest that asylum seekers are more critical of the asylum system itself as opposed to the host society, a finding similarly found in research with opponents of asylum seeking (Goodman & Speer, 2007) whose participants also constructed their opposition to asylum seeking by criticising the asylum system rather than making direct criticism of asylum seekers themselves.

What each of the extracts analysed in the present study demonstrate is that although incidents of racism may be constructed as trivial or downplayed, such a strategy may uncritically reinforce the notion of Wales as the “tolerant nation” (Williams, 2015) and that further research is needed to fully understand experiences of discrimination faced by forced migrants in Wales. The current study has shown a number of ways in which discrimination is experienced by asylum seekers and refugees living in Wales and supports the findings of Threadgold et al. (2008) and Crawley and Crimes (2009) in demonstrating the many ways in which this discrimination is a feature of life for ethnic minorities living in Wales. Indeed, the analysis of the extracts in the current study suggests that Williams (2015) may indeed be correct to challenge the tolerance thesis in light of the growing superdiversity experienced in Wales and that the more banal forms of everyday racism described by Kris and Mustafa also need to be considered.

This research also highlights problems for future research with asylum seekers and refugees in Wales and the extent to which their experience of the asylum system may, or may not, lead them to downplay potentially discriminatory experiences they have had. If discrimination is consistently played down, or constructed as trivial, as shown in the extracts presented here, it may be more difficult to identify and challenge racism in Wales, particularly the kinds of “hidden” racism constructed by Kris and Mustafa. In this paper, I have also shown the importance of the ways in which questions are constructed by the researcher in an interview context. Use of “any” questions, which Heritage and Robinson (2011) suggest are more negatively polarised, may make it more difficult for interview participants to make a denial and also require interview participants to account for such responses. Although I noted a general reluctance to make accusations of discrimination in the extracts analysed in this paper and have suggested that this may be due to not wanting to evaluate the host society negatively, there may be other considerations that are worthy of discussion. First, the researcher who undertook each of the interviews was a white British male and therefore the accounts offered by participants may have been constructed with an awareness of this. It may therefore be a case of downplaying racism in order to positively evaluate the society, of which the researcher is a member. Similarly, it would be remiss not to consider the context of the asylum system in relation to the lives of participants, whether they are still a part of that system or have recently been part of it. Indeed, for many of the participants in this study, the process of being interviewed may have been their first in the United Kingdom since their asylum interview.

## 5 CONCLUSIONS

This research has focused on the ways that refugees and asylum seekers in Wales construct accounts of their experiences of racism that they have faced within a research interview situation. The examples presented show a reluctance to make accusations of racism or discrimination. When incidents are reported, they are frequently constructed as trivial, downplayed, or explained through reference to some part of their own identity that avoids making criticisms of their host society and avoids risking their status as a person in need of protection. However, they also demonstrate that although some participants constructed accounts of explicitly racist incidents, others focused more upon more everyday and banal forms of racism that they have had to negotiate whilst living in Wales. The findings have implications for debates about Welsh “tolerance” (Williams, 2015) and for the ways in which the Welsh Government responds to these challenges.

## ENDNOTES

1 All of the names of participants given in this paper are pseudonyms to protect the identity of those who took part in this study.

2 See Appendix A for list of transcription conventions.

## APPENDIX A

### A.1

Note on transcription conventions (Potter, Tileaga, & Hepburn, 2011)

(.): Short untimed pauses
(1.0): A timed pause (in seconds)
heh heh: Voiced laughter
hhh: in‐breath
hhh: out‐breath
=: Indicates the break and subsequent continuation of a single interrupted utterance.
> <: Speech noticeable quicker than preceding talk
____: Stressed or emphasised speech
° °: Audibly quieter speech
